# Transient electromagnetic characteristics of coal seams intruded by magmatic rocks

**DOI:** 10.1371/journal.pone.0263293

**Published:** 2022-02-16

**Authors:** Yang Yang, Bin Xiong, Sanxi Peng, Siqin Liu, Hanbo Chen, Tianyu Zhang

**Affiliations:** 1 College of Earth Sciences, Guilin University of Technology, Guilin, Guangxi, China; 2 School of Geosciences and Info-Physics, Central South University, Changsha, Hunan, China; Chinese Academy of Geological Sciences, CHINA

## Abstract

The intrusion of magmatic rocks into coal seams affects the coal quality and leads to unforeseen hazards in safety of the coal mines’ production. This paper summarizes the mechanism of magmatic rocks intruding into coal seams, depicts the electromagnetic characteristics of the coal seams intruded by magmatic rocks, briefly describes the characteristics of transient electromagnetic method (TEM), and introduces the method of detection by TEM and the data processing steps. Then, the effectiveness of TEM in detecting the ranges of the coal seams intruded by magmatic rocks is theoretically analysed. It is observed that after the intrusion of magmatic rocks in the coal seams, the electromagnetic characteristics (secondary field potential and resistivity) will be dramatically affected, namely high secondary field potential and low resistivity. For experimental verification purposes, this study selects the test project of the Tongxin Minefield in the Datong Coalfield in Shanxi, China as an example, and the accuracy for the detection of the ranges of the coal seams intruded by magmatic rock using TEM is successfully verified.

## Introduction

During the production process of some coal mines, magmatic rocks’ intrusion into the coal seams has been observed [1]. The intrusion of magmatic rocks into the coal seams results in the waste of coal resources, and abolishes the quality and reduces the industrial value of coal, while also leading to many unforeseen hazards in coal mining [2–4]. Detecting the distribution of magmatic rocks intruding into the coal seams is of great practical significance for coal mine production safety.

Many scholars have performed much research regarding the detection methods of magmatic rocks’ intrusion into coal seams, and several exploration techniques have been proposed. One of these is a direct prediction method based on drilling and logging data. Dai et al. attempted to obtain the pseudo-density curve using the dual logging curve fusion method to achieve multi-parameter lithology inversion, and achieved preliminary results [5]. Liu used the resistivity curve and gamma curve to reconstruct the density curve and acoustic curve, and realized the prediction of magmatic rocks [6]. Logging curves can be used to directly show the physical properties of magmatic rocks in the vertical direction. However, conventional acoustic and density logging curves cannot clearly distinguish the difference between magmatic rocks and surrounding rocks, nor can they precisely describe the spatial distribution of magmatic rocks. Drilling data can be used to directly obtain information regarding the composition, depth, occurrence and other information of magmatic rock with high vertical resolution, but its cost is high. In addition, the network of drills will seriously affect the detection accuracy of the magmatic rock boundary, which also limits its implementation in large scale.

Another method is a geophysical prediction such as gravitational, magnetic and seismic methods. Deng et al. applied the combined inversion of gravitational and magnetic methods to effectively detect magmatic rocks in a basin area [7]; Wang et al. used magnetic method to effectively identify the intrusion boundary of magmatic rocks [8]; Sun et al. applied seismic facies analysis technology to achieve remarkable results in the delineation of magmatic rock intrusion range [9]; and Cui et al. integrated seismic inversion and seismic facies analysis methods, and achieved good results in terms of defining the scope of magmatic intrusion [10]. Although gravitational and magnetic methods can be used to detect the horizontal distribution of magmatic rocks to a certain extent, it is difficult to use them to effectively identify the depth in the vertical direction. On the other hand, the resolution of seismic data obtained by conventional seismic exploration is limited, and it is difficult to accurately obtain the magmatic rock layers intruding into the coal seams, which results in the interpretations of seismic methods not being able to fully meet the requirements.

At present, there are few relevant studies regarding the prediction of the distribution of magmatic rocks’ intrusion into coal seams using transient electromagnetic method (TEM). When the magmatic rocks intrude into the coal seams, the physical properties of the coal seams will be significantly altered, and significant differences in electrical characteristics at the interface between the magmatic rocks and coal seams will be formed. Aiming at the problem of coal seams intruded by magmatic rock, this paper analyzes the mechanism of magmatic rocks’ intrusion into coal seams and the electrical characteristics of coal seams intruded by magmatic rocks. Then, the method of detecting the magmatic rocks’ intrusion into coal seams by TEM was introduced. Combined with an engineering example, it can be found that using TEM to detect the range of coal seams intruded by magmatic rock has an ideal effect.

## Methodology

### Mechanism of magmatic rocks intruding into coal seams

**Fig 1:**
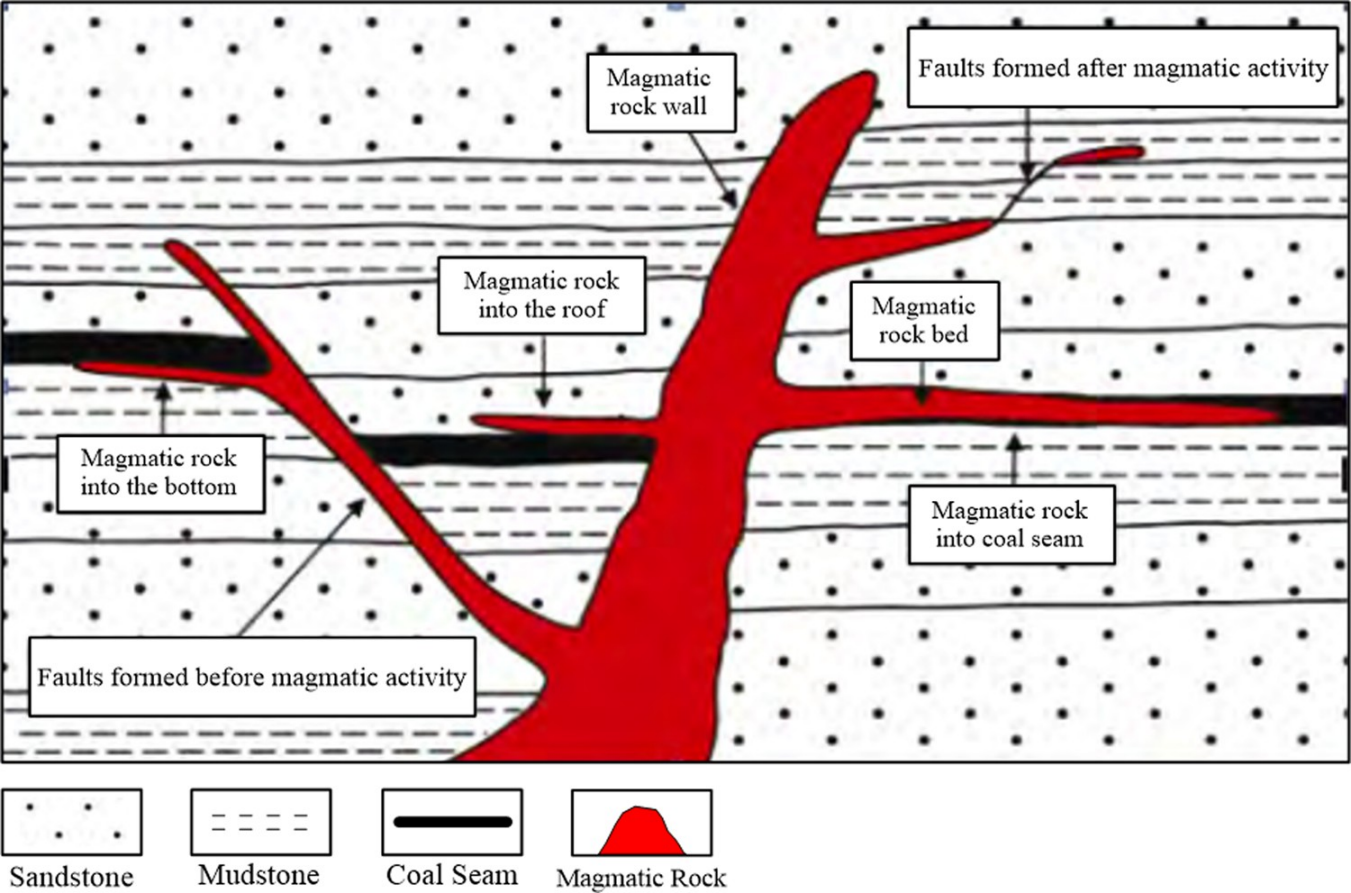
Schematic diagram of magmatic rock intruding into coal seam.

After high-temperature magma has been formed in deep underground, it flows upward along the fracture channel (faults) to reach the coal-measure strata. When the top surrounding rock resistance is greater than the uplifting pressure surrounding the magma, the magma then alters its original direction of movement, intruding from the parts with larger compressive stress to those with smaller compressive stress along the horizontal direction. Compared with the harder roof and floor layers, coal seams are generally softer and more layered, which leads to the coal seams being more susceptible to be intruded by magma. With the flowing of magma, the pressure gradually releases and the temperature decreases, then compound intrusions are eventually formed, as are rock walls and rock beds in different scales (Fig 1) [11–14].

### Electrical characteristics of coal seams intruded by magmatic rocks

Compared with coal seams, magmatic rocks have relatively low resistivity characteristics [15, 16], which are mainly caused by two reasons:

On the one hand, in the process of forming magmatic rocks by condensation of high-temperature magma, part of the gas escapes, forming a loose and porous almond structure, while some sections quickly condense to form a crystal chip and glass chip structure [17, 18]. Compared with coal seams, magmatic rocks contain more pores and fissures, in which there is much interconnected pore water. Due to the pore water’s strong conductivity, magmatic rocks’ overall resistivity is generally low [19–21]. On the other hand, the main chemical composition of magmatic rocks is SiO_2_, along with small amounts of Al_2_O_3_, Fe_2_O_3_ and other metal minerals, thus it possesses high electrical conductivity. The structural and compositional characteristics of magmatic rocks lead to their lower resistivity than the coal seams [22–24]. This forms the physical basis of the transient electromagnetic method to detect the distribution of coal seams intruded by magmatic rocks.

### Characteristics of the transient electromagnetic method

TEM is a time-domain electromagnetic induction method. The observation data have only a minor relationship with the primary field, and can be separated from the primary field in time, which is known as separability in time. Next, according to the sampling time, the secondary field signal received by a specific time node is transmitted from different exploration depths, and the secondary field signal is collected. After processing, the geological information at different depths can be obtained, which is known as separability in space [25–29]. The characteristics of TEM are based on these two separabilities. TEM possesses the following features:

1. In high resistivity surrounding rock areas, there is generally no false anomaly due to terrain influence; while in low resistivity surrounding rock areas, due to the use of multi-channel observation data, the terrain impact of the early survey track is also easy to distinguish, and the workload of terrain correction is small [30];
2. When the equivalent central loop and identical point combination mode is used for observation, the distance between the receiving system and the vertical top of the target is not far, thus the close coupling can be obtained, and the detection ability of the target is strong [31];
3. The data acquisition work is efficient and straightforward, and the requirements of coil point position and receiving distance are not highly stringent;
4. It has a strong ability to penetrate the high resistance shielding layer, and is sensitive to low resistance; in addition, it can easily highlight low resistance anomalies, such as water bodies [32].

### Data processing

Through data processing, the apparent resistivity contour map of a target layer and the secondary field potential contour map of a certain channel can be drawn. Then, based on these two maps, the data interpretation can be carried out. Below are the steps which can be adopted for data processing:

1. According to a specific observation time ***t***, the secondary field potential can be calculated by Formula (1):
$$\frac{\partial B(t)}{\partial t}=-\frac{1}{2\pi }\int_{0}^{\infty }Re[B(\omega )]\;cos\;(\omega t)d\omega$$ (1)
  where ***B*** is the magnetic induction intensity in the vertical direction of the central loop, T; ***t*** is the observation time, ms; and ***ω*** is angular frequency, rad/s.
2. According to the secondary field potential $\frac{\partial B(t)}{\partial t}$ and observation time ***t***, the apparent resistivity can be calculated by Formula (2):
$$\rho \tau (t)=\frac{\mu_{0}}{4\pi t}\times {\left(\frac{2\mu_{0}Mq}{5t\partial B(t)/\partial t}\right)}^{2/3}$$ (2)
  where ***ρτ***(***t***) is the apparent resistivity of observation time ***t***, Ω·m; ***μ***_0_ is the permeability in a vacuum, H/m; ***M*** is the transmitting magnetic moment, A/m^2^; ***q*** is the effective area of receiving coil, m^2^; ***t*** is the the observation time, ms; $\frac{\partial B(t)}{\partial t}$ is the secondary field potential, μV/A;
3. Acording to Formula (3) given in part 5.6.3.2 of the National Regulation of the People’s Republic of China “Technical specification for the transient electromagnetic method of ground magnetic source (DZ/ t0187-2016),” the detection depth ***H*** can be calculated from the observation time ***t*** and apparent resistivity ***ρτ***(***t***), and the relationship between the “apparent resistivity and time” can be transformed into the relationship between the “apparent resistivity and depth”:
$$t=\frac{H^{2}}{784\times \rho \tau (t)}$$ (3)
  At the same time, combined with Formulas (1) and (3), the target channel of the secondary field can be determined, which corresponds to the depth of the target coal seam;
4. The secondary field potential value of the target channel is picked from each surveying point, after which the secondary field potential contour map of the channel can be drawn;
5. The apparent resistivity is picked along the target coal seam, and the contour map of apparent resistivity in the target coal seam can be drawn;
6. According to the abnormal value, and combined with the known data, the abnormal range can be delineated on the contour map of the target channel secondary field potential and the contour map of the apparent resistivity of the target coal seam.

## Engineering verification

In order to verify the effectiveness of the transient electromagnetic method in detecting coal seams intruded by magmatic rocks, we carried out transient electromagnetic exploration near the 8112 working face of the Tongxin Minefield.

### Geological setting

**Fig 2:**
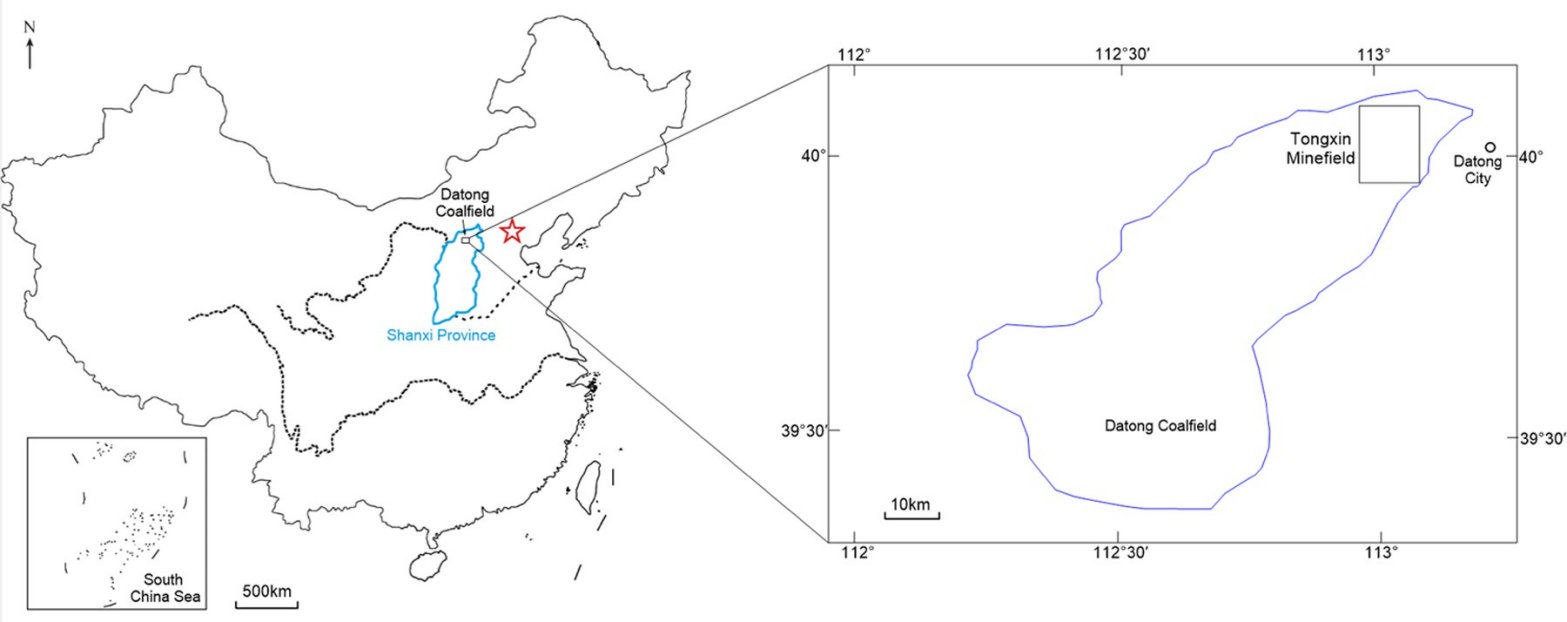
Locations of the Datong Coalfield and Tongxin Minefield.

The Tongxin Minefield is located on the northeast edge of the Datong Coalfield, Shanxi Province, China (Fig 2). The basement stratum in this area is the Jining Group of upper Archean, and all subsequent layers are successively deposited on this basis. There are more than 10 coal measure strata in the minefield. The main coal measure strata are the Jurassic Datong formation (including coal seams Nos. J3, J8, J9, J11 and J14), Permian Shanxi Formation (including coal seam No. Shan 4), Carboniferous Taiyuan Formation (including coal seams Nos. 3–5 and 8). At present, coal seam No. 3–5 is being mined.

**Fig 3:**
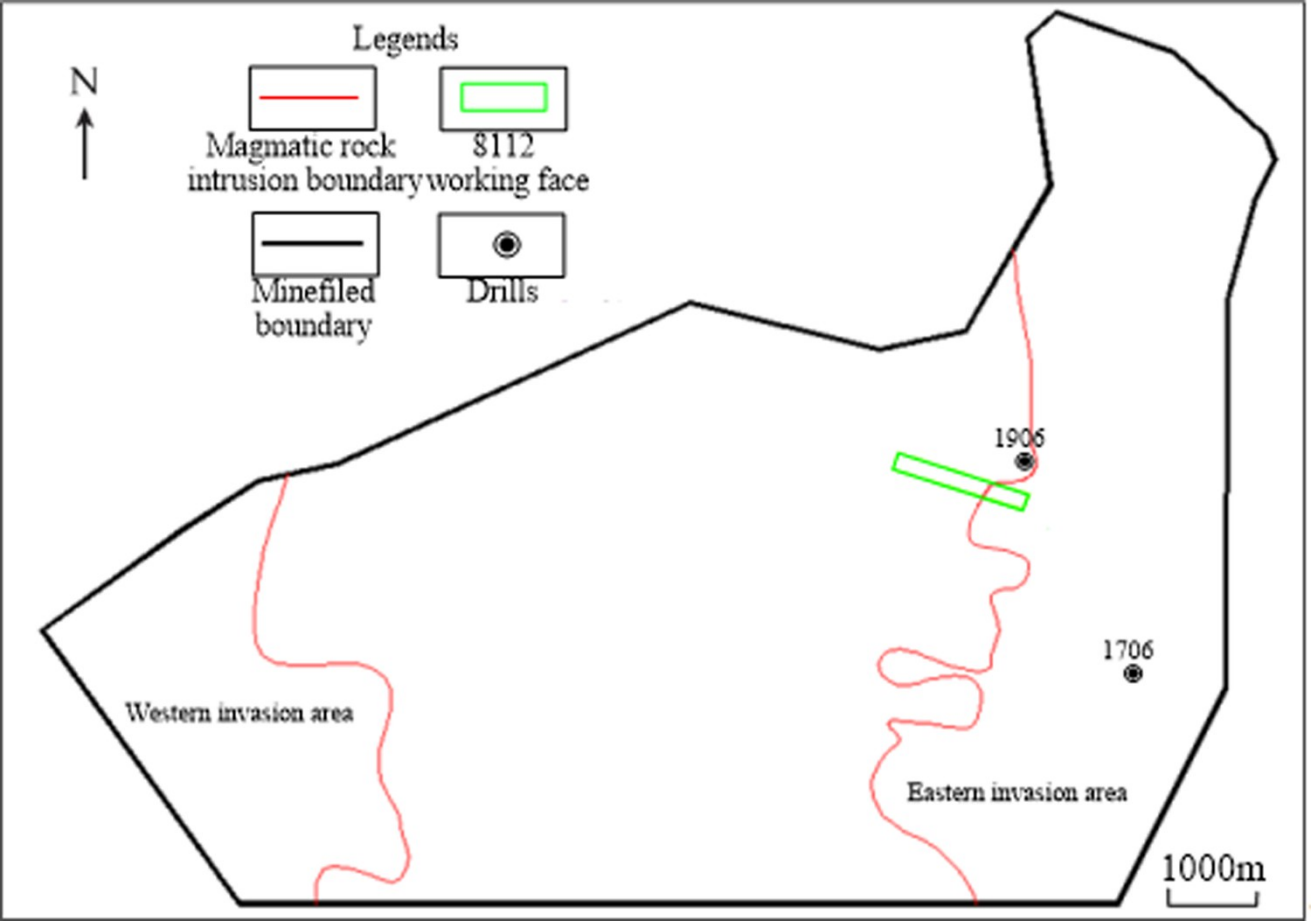
Schematic map of areas of magmatic rocks intruding into the coal seams in the Tongxin Minefield.

The Shanxi Coal Geology 115 Survey Institute and Geological Survey Department of Datong Coal Mine Group have carried out geological work such as logging, drilling and geological mapping, and have initially investigated the intrusion of magmatic rocks into coal seams since the 2000s in the Datong Coalfield near the study area [33, 34]. According to past geological data, the range of magmatic rocks’ intrusion into coal seams in the Tongxin Minefield is divided into the eastern and western parts, and the study area is near the eastern part (Fig 3). There are 31 magmatic rock drills in the minefield, including 21 in the eastern intrusion area and 10 in the western intrusion area. In the eastern intrusion area, magmatic rocks mainly intrude into coal seams No. 3–5 and 8, causing severe damage to the coal seams. In the western intrusion area, magmatic rocks mainly intrude into coal seam No. 8 and has relatively weak affects. The maximum vertical intrusion range of magmatic rocks is 75 m (drill No. 1706). Near the minefield boundary, the vertical intrusion range becomes smaller, the minimum being about 0.15 m (drill No. 1906). There are as many as 19 layers of coal seams intruded by magmatic rocks, of which the maximum thickness of a single layer is 15.9 m (drill No. 1706). In the center of the intrusion area, the magmatic rock is thicker and has many sub-layers, and gradually pinches out to the edge [35].

**Fig 4:**
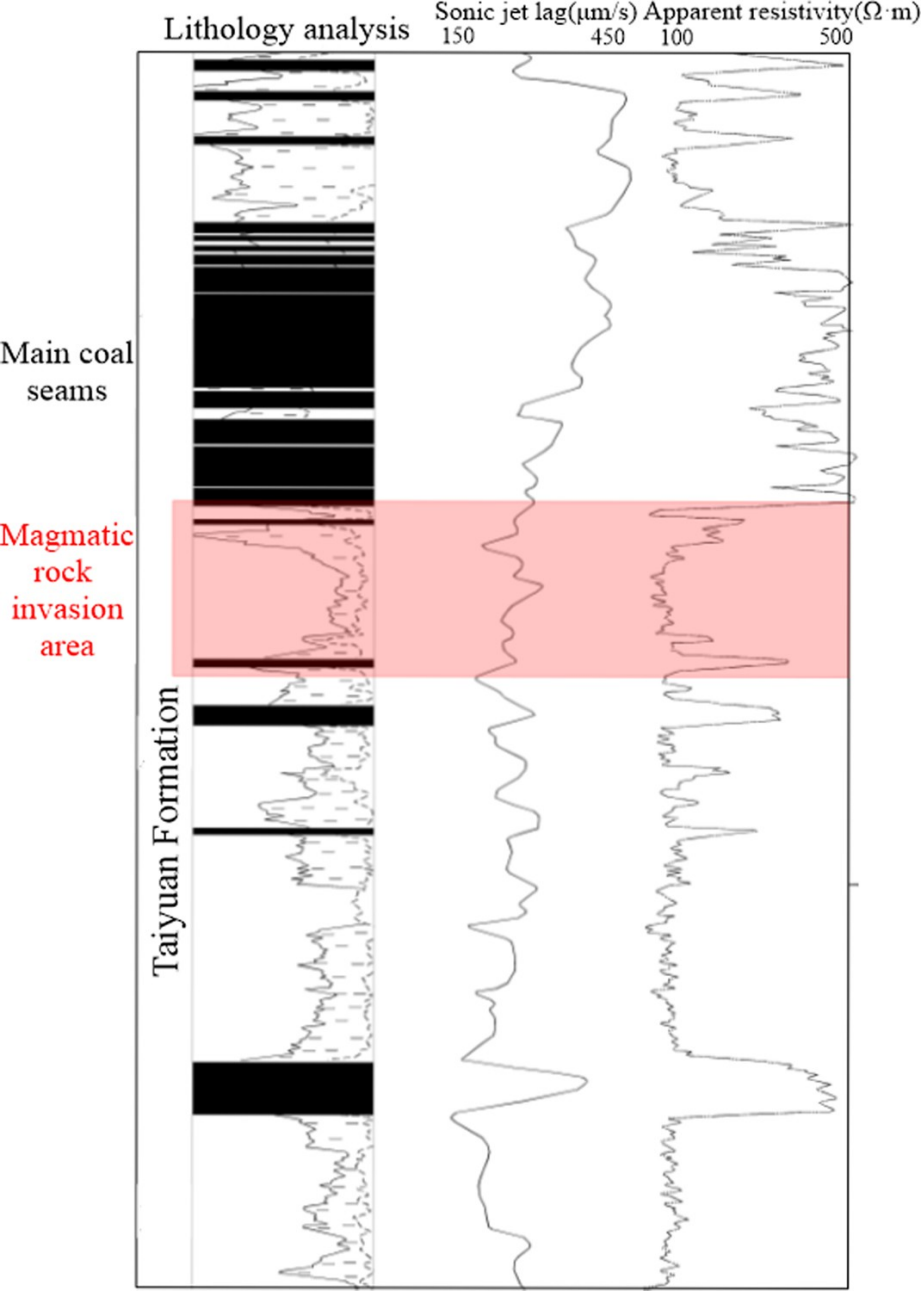
Comprehensive logging curve of the Tongxin Minefield, using lithology curve analysis.

The magmatic rocks in the Datong area are generally lamprophyre with fine-grained and massive structures. According to the study by Zhu et al. [36], the main mineral components of lamprophyre in the Datong area are biotite and potash feldspar. Fig 4 shows that the resistivity of magmatic rocks is lower than that of coal seams in the study area.

### Testing work

**Fig 5:**
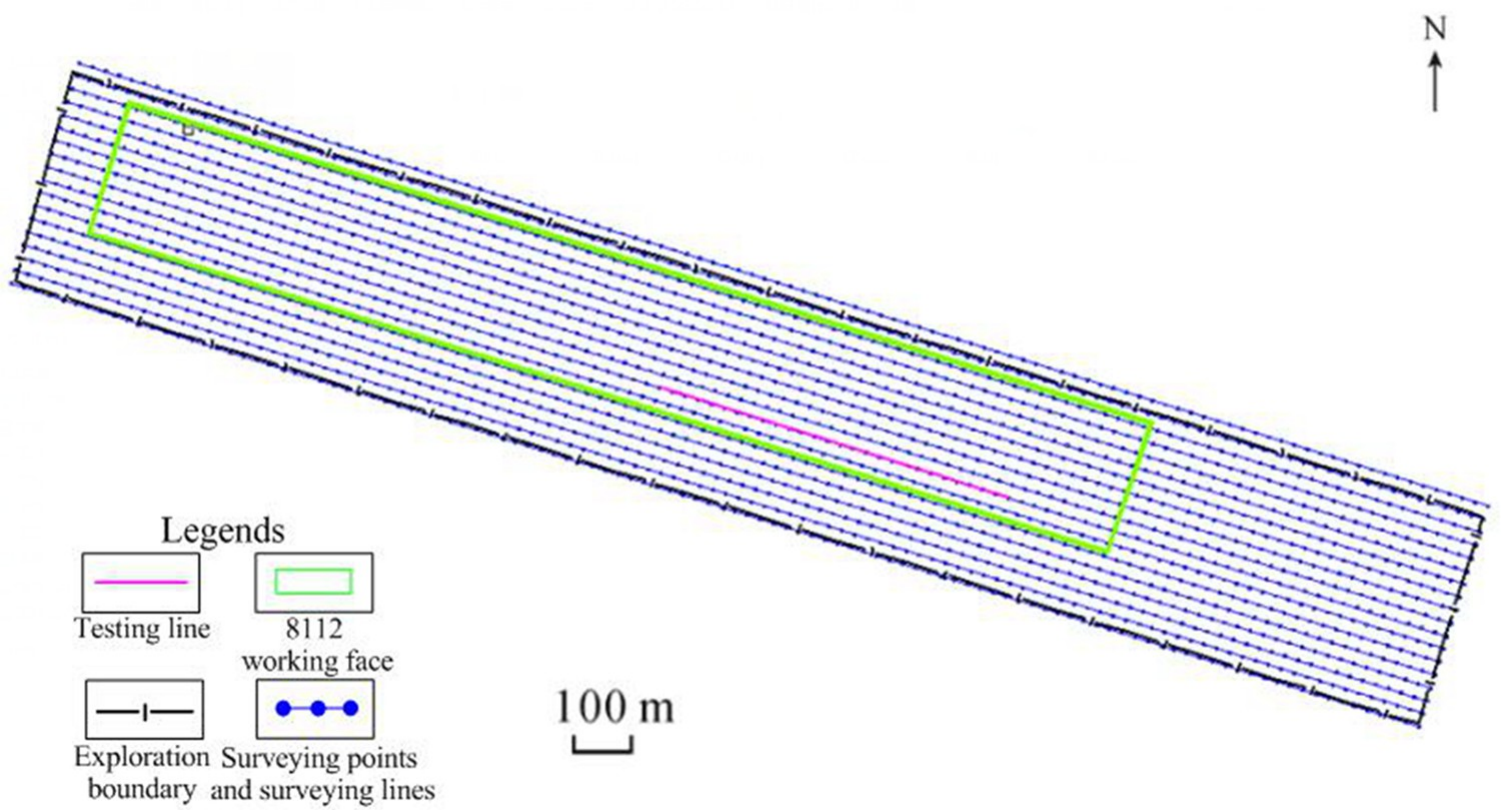
Diagram of the TEM workload and the testing line location.

There are 18 TEM surveying lines and 1980 surveying points in the study area. The dimensions of the working network are 20×20 m, i.e. the line distance is 20 m, and the point distance is 20 m, as shown in Fig 5. In the data collection work, the GDP-32II multi-function workstation produced by Zonge Co. was used by our study group during the period of June to July 2018. The transmitting parameters were as follows: the transmitting coil size was 480×480 m, the transmitting current was 16 A, and the number of turns of the transmitting coil was 1. The receiving parameters were as follows: the acquisition probe matched with GDP-32II was used to collect data, which had an equivalent acquisition area of 10,000 m^2^, and the data sampling delay time was 320 μs.

**Fig 6:**
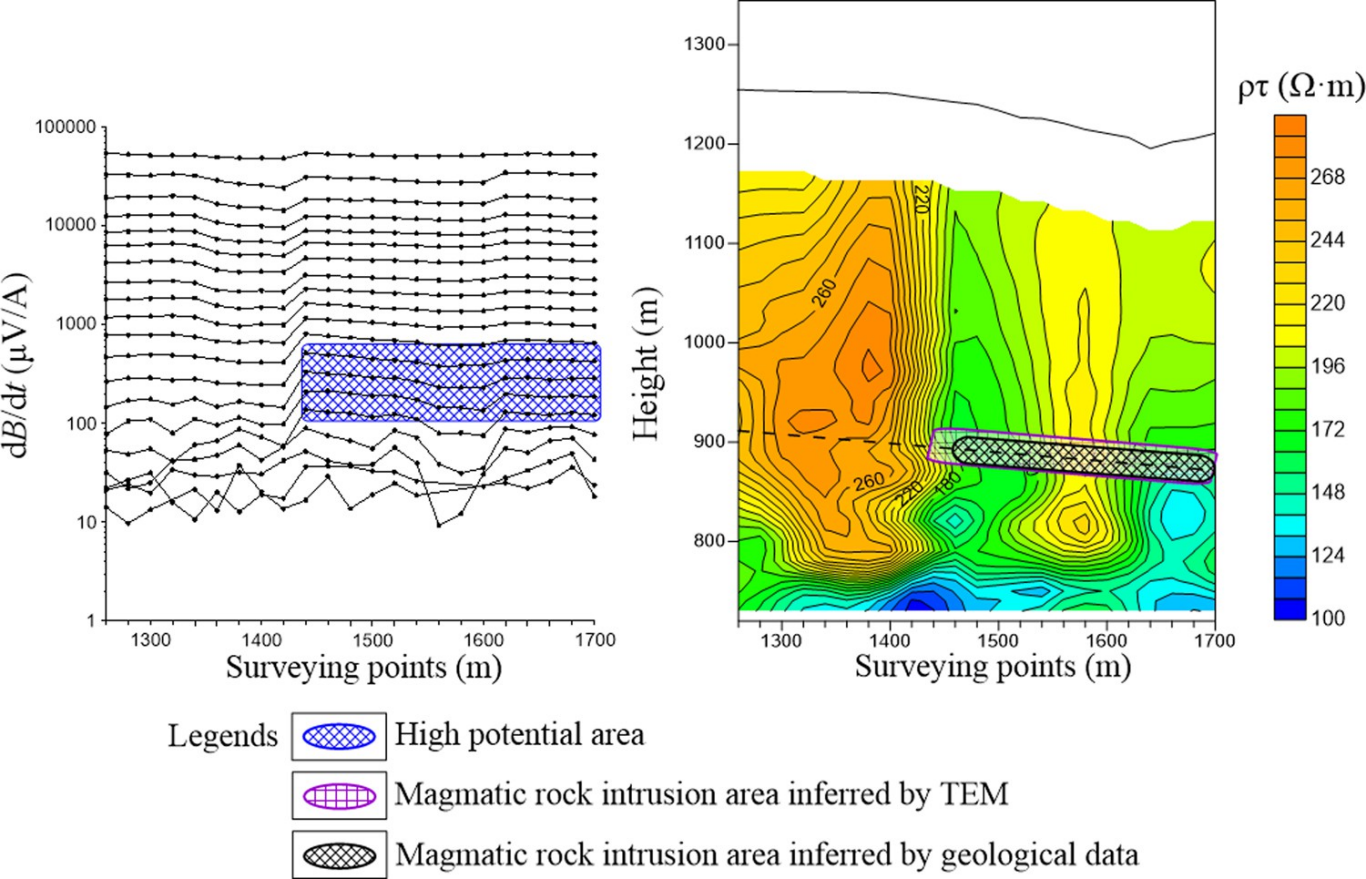
Multi-channel potential profile and apparent resistivity profile of the testing line.

According to the magmatic intrusion areas shown by geological data, the 8112 working face was located at the junction of the intrusive area and non-intrusive area. The testing work was conducted to distinguish The electrical difference between the magmatic intrusion areas and the coal seams. Fig 6 depicts the multi-channel potential profile and apparent resistivity profile of the testing line. According to the calculation result of data processing described before, the 13th channel of secondary field corresponds to the No. 3 coal seam. It can be seen that there is a high potential anomaly in the 1440–1700 section of the 13th channel, with a potential value of greater than 400 μV/A. This value is regarded as the threshold value of potential anomaly, and is approximately 30% higher than that of the normal value about 300 μV/A. In the apparent resistivity profile, significantly low apparent resistivity (magenta grid) appears in the 1440–1700 section near the coal seam No. 3–5, and the apparent resistivity value is less than 200 Ω·m, which is regarded as the threshold value of apparent resistivity anomaly. Finally, the 1440–1700 section is inferred as a magmatic intrusion area, and is in good agreement with geological data (black grid), thereby indicating that the TEM effectively detects the range of magmatic rocks’ intrusion into the coal seams.

### Application effect

**Fig 7:**
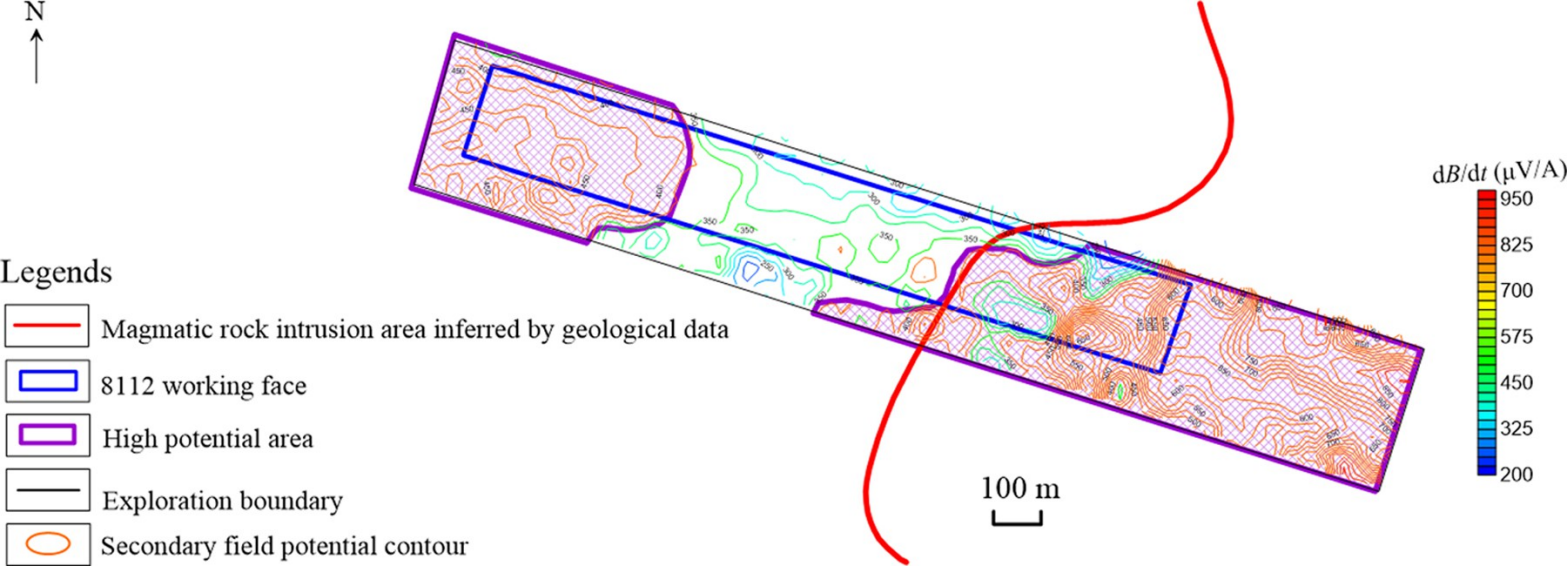
Contour map of the No. 13 channel secondary field potential.

**Fig 8:**
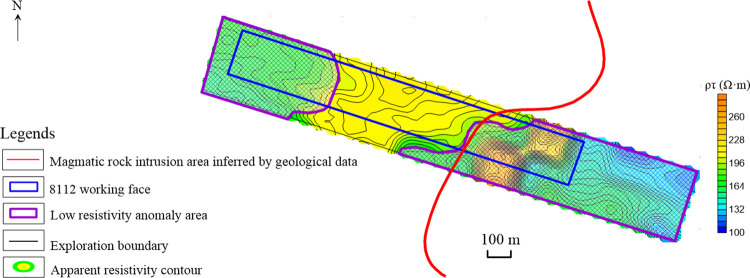
Contour map of the apparent resistivity of coal seam No. 3–5.

According to the data processing steps, the contour map of the No. 13th channel secondary field potential and the contour map of apparent resistivity of coal seam No. 3–5 are respectively drawn, as shown in Figs 7 and 8. From these two figures, it can be concluded that the coal seams intruded by magmatic rocks exhibit two electromagnetic characteristics: high potential and low resistivity.

It can be seen that the secondary field potential of the No. 13th channel in the study area is between (200–800) μV/A. According to the testing result, the areas where the secondary field potential is greater than the threshold 400 μV/A are determined as high potential abnormal areas. Two high potential abnormal areas are delineated, respectively located in the eastern and western sides. The eastern anomaly area has a more extensive range (within the magenta range in the image). Combined with the geological data, it is inferred to be the magmatic rocks’ intrusion range (Fig 7).

At the same time, the apparent resistivity in the study area varies widely, throughout the range of (120–255) Ω·m. According to the testing result, the areas where the apparent resistivity value is less than the threshold 200 Ω·m are determined to be the low resistivity abnormal areas. Similar to the contour map of the No. 13 channel secondary field potential, these areas are respectively located in the eastern and western sides. The eastern anomaly area has a large range (within the magenta range in the image). Combined with the geological data, it is inferred to be the magmatic rocks’ intrusion range (Fig 8).

### Verification

**Fig 9:**
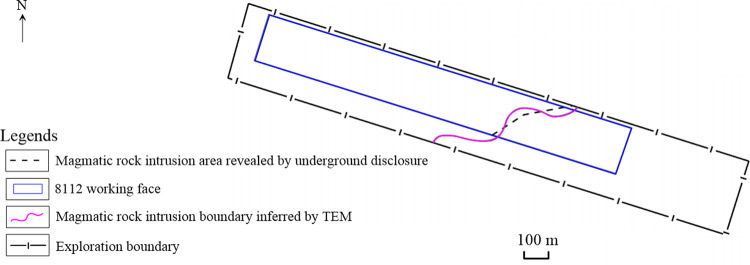
Comparison of magmatic rock intrusion revealed by underground survey with TEM detection.

In the process of roadway excavation in the 8112 working face of the Tongxin Minefield, intrusion of magmatic rocks into coal seam No.3-5 was observed. According to the actual conditions of magmatic rock intrusion revealed in the roadway, it is determined that the exposure of the roadway is fundamentally consistent with the detection of TEM detection. The minimum error of TEM detection boundary is approximately 11 m, and the maximum error is about 30 m, as shown in Fig 9.

## Conclusion

This paper proposes a method for detecting the range of coal seams intruded by magmatic rock by using the transient electromagnetic method (TEM). Combined with the analysis of engineering examples, the following conclusions can be drawn: After the coal seams have been intruded by magmatic rocks, the physical properties will alter greatly. It is important to use geological and logging data to analyze the physical characteristics of the coal seams intruded by magmatic rock, which is of guiding significance for the conduction of the transient electromagnetic method. According to the geological data, the intrusion area of magmatic rocks can be macroscopically delineated. Based on this, TEM can be used to detect the precise range of magmatic rock intrusion into coal seams. It can also be observed that there is a significant electrical difference between the magmatic rocks and normal coal seams. The electrical characteristics of coal seams intruded by magmatic rocks are low resistivity and high secondary field potential. The results reveal that the effect of using TEM to detect the range of coal seams intruded by magmatic rocks is satisfactory.

## Supporting information

S1 Data (RAR)

## References

[1] Chen YL, Qin Y, Ji M, Duan HF, Wu CF, Shi QM, et al. Influence of lamprophyre sills on coal metamorphism, coalbed gas composition and coalbed gas occurrence in the Tongxin Minefield, Datong Minefield, China. International Journal of Coal Geology. 2019; Volume 217: 103286.

[2] Cooper JR, Crelling JC, Rimmer SM, Whittington AG. Coal metamorphism by igneous intrusion in the Raton Basin, CO and NM: Implications for generation of volatiles. International Journal of Coal Geology. 2007; 71(1): 15–27.

[3] Gurba LW, Weber CR. Effects of igneous intrusions on coalbed methane potential, Gunnedah Basin, Australia. International Journal of Coal Geology. 2001; 46(2–4): 113–131.

[4] Jiang MM, Liu GJ, Wu B, Zheng LG. Geochemistry of rare earth elements(REEs) in coal from magmatic intrusion area from Wolonghu Coal Mine. Journal of University of Science and Technology of China. 2012; 42(1): 10–16.

[5] Dai FY, Cui RF, Chen TG. The application of multiple parameters lithological inversion to coalfield seismic exploration. Geophysical and Geochemical Exploration. 2013; 37(1): 104–107.

[6] Liu P. Prediction of magmatic intrusion by using quasi-density inversion technique based on lithological columnar data reconstruction: Taking Mining Area 103 of Qinan Coal Mine as an example. Chinese Journal of Engineering Geophysics. 2019; 16(4): 500–507.

[7] Deng RL, Li QH, Song GQ, Liu TY. Investigation on distribution of igneous rock in Bayabhaote Basin with joint inversion and integrated interpretation of gravity, magnetic and MT data. Geophysical Prospecting for Petroleum. 2016; 41(2): 222–225.

[8] Wang ZK, Peng DR, Lu L, Gou JC. Detection of magmatic intrusive mass by geophysical methods in coal mining area. Coal Geology & Exploration. 2015; 43(1): 91–95.

[9] Sun XK, Cui RF. Application of seismic faces analysis in detecting the magmatic intrusion zones. Coal Geology & Exploration. 2010; 38(5): 58–61.

[10] Cui DW, Yu JC, Dai FY. Seismic interpretation method for the magmatic intrusion extent in coal seams. Coal Geology & Exploration. 2014; 42(2): 76–79.

[11] Lister JR, Kerr RC. Fluid-mechanical models of crack propagation and their application to magma transport in dykes. Journal of Geophysical Research. 1991; 96: 10049–10077.

[12] Rimmer SM, Yoksoulian LE, Hower JC. Anatomy of an intruded coal, I: Effect of contact metamorphism on whole-coal geochemistry, Springfield (No. 5) (Pennsylvanian) coal, Illinois Basin. International Journal of Coal Geology. 2009; 79(3): 74–82.

[13] Menand T, Tait SR. The propagation of a buoyant liquid fissure from a source under constant pressure: An experimental approach. Journal of Geophysical Research Solid Earth. 2002; 107: 16–14.

[14] Menand T. The mechanics and dynamics of sills in layered elastic rocks and their implications for the growth of laccoliths and other igneous complexes. Earth & Planetary Science Letters. 2008; 267: 93–99.

[15] Fu DG, Zhou YM, Zhang CQ, Chen QG, Qin XP. Geological characteristics of lamprophyres and their relations with gold mineralization of the Xiaoshuijing Gold Deposit in central Yunnan Province. Geology and Exploration. 2010; 46(3): 414–425.

[16] Wang L, Tang DZ, Xu H, Li S, Pang JD, Yao CH, et al. Magmatism effect on different transformation characteristics of coal reservoirs physical properties in Xishan coalfield. Journal of China Coal Society. 2015; 40(8): 1900–1910.

[17] Guo NX, Chen YC, Li XQ, Chen ZH, Zhao Z. Mineralogical characteristics of the granitoid exposed in the Nanling Scientific Drill Hole and implications for magmatism and mineralization in the Yinkeng Orefield, Southern Jiangxi Province. Geology in China. 2016; 43(5): 1645–1665.

[18] Liu J, Zhou Y, Xie DG, Ji XX, Li DW. Geochronology, geochemistry and geological implications of the lamprophyre in Jianshui, East Yunnan Province. Geology in China. 2016; 43(6): 1977–1991.

[19] Chen B. Experimental study on concealed reservoir thermal structure in magmatic area of northern Fujian by means of comprehensive geophysical exploration. Chinese Journal of Engineering Geophysics. 2018; 15(6): 798–803.

[20] Hoskin PWO, Schaltegger U. The composition of zircon and igneous and metamorphic petrogenesis. Reviews in Mineralogy and Geochemistry. 2003; 53(1): 27–62.

[21] Wang L, Yang LW, Wang RX, Gao J, Sun YM. Disaster occurred mechanism and prevention of coal spontaneous combustion in goaf of seam under magmatic rock bed. Coal Science and Technology. 2019; 47(1): 125–131.

[22] Hu B, Jia ZY, Zhang GB, Zhang G, Zhang CR, Sun RB, et al. Three-dimensional inversion of gravity and magnetic data and its application in the study on the characteristics of magmatic rocks in the Gangdise belt and adjacent areas, Tibetan Plateau. Chinese Journal of Geophys. 2019; 62(4): 1362–1376.

[23] Kang TH, Dong D, Wei JP, Xue WL. Experimental study on the relationship between coal resistivity and gas content. Geology and Exploration. 2016; 52(5): 918–923.

[24] Yuan H, Luo XR, Li WY, Chen W. Geochemical characteristics and tectonic significance of lamprophyre in the Gudui area of Tibet. Geology and Exploration. 2017; 53(2): 300–309.

[25] Li H, Xue GQ, Zhao P, Zhou NN, Zhong H. Inversion of arbitrary segmented loop source TEM data over a layered earth. Journal of Applied Geophysics. 2016; 128: 87–95.

[26] Li H, Xue GQ, Zhou NN, Chen WY. Appraisal of an Array TEM Method in Detecting a Mined-Out Area Beneath a Conductive Layer. Pure and Applied Geophysics. 2015; 172(10): 2917–2929.

[27] Zhou NN, Xue GQ, Hou DY, Lu YF. An investigation of the effect of source geometry on grounded-wire TEM surveying with horizontal electric field. Journal of Environmental and Engineering Geophysics. 2018; 23(1):143–151.

[28] Li H, Xue GQ, Zhang LB. Accelerated Bayesian inversion of transient electromagnetic data using MCMC subposteriors. IEEE Transactions on Geoscience and Remote Sensing. 2020; 99: 1–11.

[29] Li H, Xue GQ, Zhao P. A new imaging approach for dipole-dipole time-domain electromagnetic data based on the q-transform. Pure and Applied Geophysics. 2017; 174(10): 3939–3953. doi: 10.1007/s00024-017-1603-1

[30] Li B, Chao DC, Wei MJ, Li YF, Luo ZZ, Shang JG. The application of electromagnetic sounding method to deep iron ore exploration: A case study of the Wuyang Iron Mining Area of Henan. Geology in China. 2013; 40(5): 1644–1654.

[31] Yu M. Application of the transient electromagnetic(TEM) technology to the Keketale Pb-Zn deposit in Xinjiang. Geology and Exploration. 2013; 49(4): 723–730.

[32] Xue GQ, Yu JC. New development of TEM research and application in coal mine exploration. Progress in Geophysics. 2017; 32(1): 319–326. doi: 10.6038/pg20170145

[33] Niu XL, Feng GY, Liu F, Yang JS. Genetic study upon the platinum-group element geochemistry of the Triassic lamprophyres in the Datong area(Shanxi Province), northern margin of the North China Craton. Acta Petrologica Sinica. 2017; 33(12): 3897–3908.

[34] Liu DN, Zhou AC, Chang ZG. Geochemistry characteristics of major and rare earth elements in No. 8 raw and weathered coal from Taiyuan Formation of Datong coalfield. Journal of China Coal Society. 2015; 40(2): 422–430.

[35] Xin CH. Study on magmatic rocks distribution law in Northern Datong Coalfield. Coal Science and Technology. 2013; 41(Sup): 166–169.

[36] Zhu WP, Liu DG, Song XX. Study on Igneous Rock Petrologic Features in Datong Coalfield. Coal Geology of China. 2020; 32(6): 22–26+35.

